# Physical performance discriminating winning and losing in UEFA Champions League: a full-season study

**DOI:** 10.5114/biolsport.2025.139076

**Published:** 2024-05-07

**Authors:** Toni Modric, Sime Versic, Igor Jukic, Damir Sekulic

**Affiliations:** 1 Faculty of Kinesiology, University of Split, Split, Croatia; 2 High Performance Sport Center, Croatian Olympic Committee, Zagreb, Croatia; 3 Faculty of Kinesiology, University of Zagreb, Zagreb, Croatia

**Keywords:** Match running performance, Physical demands, Match outcome, Playing positions, Contemporary soccer

## Abstract

This study aimed to examine the differences in physical performance when winning and losing in UEFA Champions League (UCL) matches. Data were collected using an optical tracking system from all UCL matches (n = 125) in the 2022/23 season. A linear mixed model was used to examine the differences in physical performance in won and lost matches while controlling for match location, team formation, opponent quality, playing time, red cards, and between-player, -team, and -match variations. The results indicated that (i) wingers and forwards covered ~ 20% greater high-intensity running distance in won compared to lost matches (Cohen’s d (d) = 0.76 and 0.96, respectively), (ii) central defenders and fullbacks covered ~ 15% greater high-intensity running distance in lost compared to won matches (d = 0.59 and 0.32, respectively), (iii) offensive midfielders achieved ~ 2% greater low-intensity running distance (d = 0.59) while defensive midfielders performed ~ 2% greater total distance (d = 0.81) in won compared to lost matches. These findings suggest that winning was physically more demanding for offensive players but losing was more demanding for defensive players, while midfield players’ physical demands were similar irrespective of winning and losing. Soccer coaches should consider implementing an extended period of recovery for offensive and defensive players following won and lost matches, respectively.

## INTRODUCTION

Determining the success criteria for soccer teams has been one of the prominent research topics in the scientific community in recent years [1, 2]. One of the main goals of sports science is to clarify the strategic performance objectives of teams and examine the indicators that improve their competitive outcomes [3, 4]. Thus, sport scientists and soccer coaches have regularly attempted to quantify the match performance of players to determine how the game was won or lost [5, 6]. So far, the greatest focus has been on technical-tactical performances such as ball possession, passes, crosses and/or shots [7–9]. However, match performance in soccer is not only determined by technical-tactical performances but is highly dependent on the interactions between technical-tactical and physical performance [10].

Although players’ technical-tactical performance is decisive for winning matches [11], achieving high levels of technical-tactical performance might not be possible without an appropriate level of physical performance [12]. For example, successful invasion in the last third of the pitch by forwards, efficient counterattacks by wingers, offensive crossing by fullbacks, or defensive transition by central midfielders and central defenders require a spatio-temporal advantage over the opponent players. As this can done only by running at higher speeds, physical performance, especially high-intensity activities, seems to be an important component of won matches [13, 14, 15, 16, 17].

It is therefore not surprising that researchers have expended great effort in providing knowledge about physical performance when winning and losing. Despite the large body of evidence [5, 6, 18, 19], the literature remains equivocal. Briefly, older studies reported fewer high-intensity activities in won matches compared to lost matches [18]. More recent studies revealed no differences in high-intensity activities regardless of the match outcome [19]. Some studies even indicated that players in specific playing positions (i.e., wide midfielders and forwards) performed greater distances at higher speeds in won matches than in lost matches [5, 6].

Although previous studies provided valuable knowledge about physical performance discriminating winning and losing, it should be taken into account that all of them analyzed physical performance obtained from only one country [5, 6, 18, 19]. Therefore, the results were undoubtedly influenced by geographical, cultural, historical, and social aspects of the observed competition [20]. Furthermore, most of these studies utilized simple methodology without controlling for the influence of various contextual factors such as team formation, match location, and opponent quality [18], which have been shown to affect physical performance. In addition, no studies have investigated physical performance discriminating winning and losing while controlling for natural match-to-match variability, players’ and/or teams’ multiple observations, players’ dismissal from the match (i.e., red cards), and total playing time in single matches [21–23]. Finally, as longitudinal analysis of football matches indicates that physical performance has changed tremendously over the last decade [24], some findings from previous studies are most likely limited in current application. Consequently, true knowledge about physical demands when winning and losing in contemporary soccer is constrained.

Considering all previous limitations, we believe that new research utilizing a more complex methodological approach and more recent data by analysing multiple teams from different countries is warranted. One of the most elite competitions that includes teams from different countries is the UEFA Champions League (UCL) [25]. However, no studies have investigated physical performance profiles when winning and losing in the UCL so far. The results from research analysing physical performance profiles when winning and losing in UCL matches will provide novel findings that may be crucial to developing specific game strategies and training designs in elite-level soccer [26]. Therefore, this study aimed to examine the differences in physical performance when winning and losing in UCL matches while controlling for situational factors.

## MATERIALS AND METHODS

### Match data

Physical performance data were collected using an optical tracking system (Player & Ball Tracking System, Hawk-Eye Innovations Limited, Basingstoke, England). The reliability of the system has previously been tested using the official Fédération Internationale de Soccer Association (FIFA) test protocol for Electronic and Performance Tracking Systems (EPTS). This included comparison of data to the Vicon system (Vicon Motion Systems, Oxford Metrics, UK) at five velocity bands (0–7 km/h, 7–15 km/h, 15–20 km/h, 20–25 km/h, and 25+ km/h). The system successfully passed this test protocol (authorization number: 1015068), demonstrating a high level of reliability (a detailed report is available on the official FIFA webpage: https://inside.fifa.com/api/resource-hub/test-report?id=a50993dbc57440dab1b606abc5717e00).

### Match analysis and players’ data

Players’ physical performances were obtained from all UCL matches (n = 125) in the 2022/23 season. Two matches were preliminarily excluded from further analysis due to bad data. As this study aimed to examine the differences in physical performance when winning and losing, all matches that finished as a draw (i.e., 90 minutes plus injury time) were excluded from the analysis (n = 25). Due to methodological reasons, only the results of players who participated in the whole match were analysed, while goalkeepers were not included in the analysis due to the specificity of the position [6]. Considering that positional interchanges may influence physical performances [21], players who changed their tactical roles were excluded from the analysis. As a result, the final sample included 1087 match observations, which were classified automatically into six positional subsets by the data provider based on the players’ tactical role in the team: central defender (CD; n = 375), fullback (FB; n = 226), defensive midfielder (DM; n = 133), offensive mid-fielder (OM; n = 157), winger (WM; n = 110) and forward (FW; n = 86). Players’ identities were anonymized following the principles of the Declaration of Helsinki to ensure confidentiality. The investigation was approved by the local university ethics board.

### Variables

Physical performance variables included cumulative and relative total distance covered (TD and RTD, respectively), low-intensity running (LIR and RLIR, respectively) (< 15 km/h), moderate-intensity running (MIR and RMIR, respectively) (15–20 km/h), and high-intensity running (HIR and RHIR, respectively) (> 20 km/h) [27]. Situational variables included the six-level categorical variable “playing position”, the eight-level categorical variable “team formation”, the two-level categorical “red card”, and the continuous variables “quality of the opponent” and “playing time”. Playing position classification was done based on players’ activity on the pitch and the primary area where this activity was performed and included CD, FB, DM, OM, WM, or FW. Match location was recorded as playing at “home” or “away”. Team formation classification was done based on teams’ average positioning on the pitch during the matches which were defined by the data provider, and included the following formations: 3-2-4-1, 3-4-3, 3-5-2, 4-2-3-1, 4-3-3, 4-4-2-flat, 4-4-2-diamond, and 5-4-1. Opponent quality was evaluated using UEFA season club coefficients [28]. The red card variable was evaluated with “yes” or “no” depending on whether the match included a red card. Playing time was evaluated by the total duration of regular time plus injury time (i.e., 90 minutes plus time added by referees). Finally, to evaluate differences in physical performance between winning and losing, the three-level categorical variable “match out-come” was created, and coded as “won”, “lost” or “draw” depending on the outcome of the match after regular time (i.e., draw outcomes were later excluded from analysis).

### Statistical analysis

A linear mixed model was used to examine differences in physical performance when winning and losing. The physical variables were included as dependent variables in the model, and playing position, match outcome, match location, quality of the opponent, team formation, red card, and playing time were the independent variables included as fixed effects. Due to the hierarchical design of data, players, teams, and matches were modelled as random effects. The assumptions of homogeneity and normal distributions of residuals were verified. Cohen’s d (d) was used to identify effect sizes (ES) and interpreted as follows: trivial (< 0.2), small (≥ 0.2–0.5), moderate (≥ 0.5–0.8), and large (> 0.8) [29]. All analyses were performed using SPSS software version 25.0 (IBM-SPSS, New York, USA). Statistical significance was set at p < 0.05.

## RESULTS

Tables 1 and 2 present differences in physical performance between won and lost matches while controlling for the influence of situational factors. CDs and OMs covered more LIR (d = 0.36 and 0.59, respectively) and RLIR (d = 0.36 and 0.60, respectively) in won compared to lost matches. Also, CDs covered less HIR and RHIR in won than in lost matches (d = 0.59 and 0.58, respectively). DMs covered more TD and RTD in won compared to lost matches (d = 0.81 and 0.44, respectively). FBs covered more LIR and less HIR in won than in lost matches (d = 0.42 and 0.32, respectively). In addition, FBs had more RLIR and less RHIR in won compared to lost matches (d = 0.42 and 0.32, respectively). FWs covered more HIR and RHIR in won than in lost matches (d = 0.76 and 0.78, respectively). WMs covered more TD and HIR in won than in lost matches (d = 0.63 and 0.96, respectively). In addition, WMs had more RTD and less RHIR in won compared to lost matches (d = 0.61 and 0.96, respectively).

**TABLE 1:** Descriptive statistics and differences in cumulative physical performance in won and lost matches.

		Lost	Won	ML: home	TF: 3241	TF: 343	TF: 352	TF: 4231	TF: 433	TF: 442F	TF: 442D	OQ	PT	RC: No

	(m)	Mean ± SD		Statistics (t)										
CD	TD	10093 ± 106	10203 ± 103	0.74	–1.14	–0.44	–0.15	–0.36	–0.56	0.62	–0.61	1.95	5.90	1.62
	LIR	**8007 ± 92**	**8260 ± 89**	0.15	–3.34^*^	–0.54	–0.53	–0.36	–0.55	0.77	–0.48	–0.74	3.45	1.03
	MIR	1412 ± 74	1388 ± 70	0.04	3.02^*^	0.53	0.28	0.97	0.58	0.88	0.64	2.32^*^	1.47	–0.09
	HIR	**681 ± 26**	**582 ± 25**	1.35	1.17	0.83	0.95	–0.67	–0.15	–1.23	–0.27	–0.15	0.33	–0.79

OM	TD	11568 ± 130	11754 ± 129	–0.80	–0.83	–0.55	–1.08	–1.09	–1.33	–0.39	–0.44	3.78^*^	2.38^*^	1.63
	LIR	**8525 ± 98**	**8725 ± 94**	–1.51^*^	–3.39^*^	–0.74	–0.81	–1.75	–1.03	–0.11	–1.23	1.37	1.21	0.74
	MIR	2123 ± 90	2089 ± 87	–0.18	3.15^*^	0.93	–0.36	0.35	0.05	0.84	0.69	3.43^*^	0.83	0.97
	HIR	907 ± 45	948 ± 44	0.99	0.24	–0.36	0.09	0.96	–0.42	–0.85	0.80	0.23	1.63	0.19

DM	TD	**11267 ± 170**	**11507 ± 180**	0.35	0.41	–4.12^*^	0.66	0.96	0.33	1.91	/	1.64	0.85	0.15
	LIR	8221 ± 170	8434 ± 180	0.47	–2.37^*^	–2.77^*^	0.43	0.27	0.06	1.14	/	–0.91	0.95	1.06
	MIR	2201 ± 200	2218 ± 215	–0.45	1.77	–0.51	–0.13	0.19	–0.33	0.50	/	1.93	–0.70	–0.26
	HIR	785 ± 53	790 ± 56	–0.71	1.66	0.48	0.44	1.95	0.82	1.41	/	0.77	0.36	–0.20

FB	TD	10724 ± 108	10776 ± 108	–0.32	/	–0.86	–0.66	–0.22	–0.93	–0.76	–0.90	0.98	3.55^*^	2.58^*^
	LIR	**8034 ± 81**	**8246 ± 81**	–1.09	/	–0.50	–0.03	0.87	0.31	0.87	–0.34	0.85	2.47^*^	2.82^*^
	MIR	1578 ± 54	1520 ± 54	–0.47	/	0.07	–0.59	–0.13	–0.88	–0.82	–0.64	1.13	1.09	0.26
	HIR	**1081 ± 36**	**1007 ± 36**	1.55	/	–0.87	–0.74	–1.87^*^	–1.49	–2.85^*^	–1.19	–1.24	2.45^*^	–0.36

FW	TD	10281 ± 225	10589 ± 200	0.16	0.76	1.62	1.22	2.53^*^	1.36	2.40^*^	/	0.93	3.38^*^	2.37^*^
	LIR	7875 ± 165	7885 ± 145	0.02	–2.25^*^	0.57	0.93	1.77	0.71	1.40	/	–0.72	4.03^*^	1.85
	MIR	1589 ± 125	1687 ± 109	0.13	4.85^*^	1.00	0.33	1.68	1.07	1.56	/	0.35	–0.55	0.73
	HIR	**784 ± 57**	**943 ± 49**	0.21	0.51	1.55	0.37	2.57^*^	1.69	2.07^*^	/	1.15	0.23	1.65

WM	TD	**10671 ± 245**	**11057 ± 244**	0.11	–0.93	0.08	/	–0.50	–0.62	–0.20	/	1.80^*^	0.29	2.89
	LIR	8038 ± 218	7979 ± 212	1.38	–2.00^*^	–0.90	/	–1.16	–0.88	–0.58	/	–2.08	1.23	2.55
	MIR	1731 ± 179	1834 ± 170	–0.47	1.58	1.10	/	0.54	0.19	0.74	/	3.15^*^	–0.53	1.33
	HIR	**926 ± 64**	**1137 ± 63**	–0.66	–0.17	0.56	/	0.49	–0.04	–0.04	/	3.15^*^	–0.88	–0.52

Note: CD – central defenders, OM – offensive midfielders, DM – defensive midfielders, FB – fullbacks, FW – forwards, WM – wingers; ML – match location, TF: 3241 – team formation 3-2-4-1; TF: 343 – team formation 3-4-3; TF: 352 – team formation 3-5-2; TF: 4231 – team formation 4-2-3-1; TF: 433 – team formation 4-3-3-, TF: 442F – team formation 4-4-2-flat, TF: 442D – team formation 4-4-2-diamond, OQ – quality of opposition, PT – playing time, RC – red card; TD – total distance, LIR – low-intensity running, MIR – moderate-intensity running, HIR – high-intensity running;

* – significant influence of fixed effect at p < 0.05. Bold text denotes significant differences in physical performance in won and lost matches at p < 0.05. Reference team formation for CD, OM, FB, and WM is 5-4-1. Reference formation for DM and FW is 4-4-2-D.

**TABLE 2:** Descriptive statistics and differences in relative physical performance in won and lost matches.

		Lost	Won	ML: home	TF: 3241	TF: 343	TF: 352	TF: 4231	TF: 433	TF: 442	TF: 442D	OQ	PT	RC: No

	(m/min)	Mean ± SD		Statistics (t)										
CD	RTD	105.14 ± 1.1	106.33 ± 1.07	0.74	–1.18	–0.41	–0.14	–0.32	–0.55	0.66	–0.63	1.98^*^	–3.82^*^	1.68
	RLIR	**83.43 ± 0.96**	**86.07 ± 0.93**	0.15	–3.36^*^	–0.51	–0.51	–0.33	–0.52	0.80	–0.48	–0.75	–3.15^*^	1.06
	RMIR	14.7 ± 0.77	14.47 ± 0.74	0.04	3.03^*^	0.54	0.29	0.98	0.59	0.88	0.64	2.33^*^	0.29	–0.07
	RHIR	**7.09 ± 0.27**	**6.07 ± 0.26**	1.33	1.16	0.82	0.93	–0.69	–0.19	–1.26	–0.30	–0.14	–1.52	–0.82

OM	RTD	120.8 ± 1.36	122.71 ± 1.35	–0.79	–0.88	–0.60	–1.12	–1.14	–1.38	–0.44	–0.49	3.76^*^	–3.43^*^	1.64
	RLIR	**88.99 ± 1.02**	**91.08 ± 0.98**	–1.47	–3.43^*^	–0.76	–0.83	–1.78	–1.05	–0.14	–1.27	1.32	–3.68^*^	0.77
	RMIR	22.19 ± 0.95	21.82 ± 0.91	–0.20	3.17^*^	0.93	–0.35	0.34	0.05	0.84	0.69	3.45^*^	–0.39	0.94
	RHIR	9.48 ± 0.47	9.88 ± 0.46	0.97	0.29	–0.35	0.09	0.96	–0.42	–0.87	0.81	0.21	0.40	0.22

DM	RTD	**117.53 ± 1.79**	**120 ± 1.89**	0.33	0.44	–4.16^*^	0.64	0.99	0.32	1.89	/	1.54	–4.66^*^	0.23
	RLIR	85.78 ± 1.79	87.99 ± 1.89	0.47	–2.35^*^	–2.78^*^	0.44	0.29	0.07	1.16	/	–0.94	–2.09^*^	1.07
	RMIR	22.94 ± 2.1	23.11 ± 2.25	–0.45	1.79	–0.52	–0.13	0.20	–0.32	0.48	/	1.91	–1.33	–0.23
	RHIR	8.18 ± 0.55	8.24 ± 0.59	–0.71	1.68	0.46	0.44	1.96	0.82	1.38	/	0.74	–0.65	–0.16

FF	RTD	112.1 ± 1.14	112.67 ± 1.13	–0.28	/	–0.92	–0.71	–0.26	–0.98	–0.77	–0.98	0.97	–4.32^*^	2.56^*^
	RLIR	**83.97 ± 0.86**	**86.2 ± 0.86**	–1.05	/	–0.54	–0.06	0.84	0.28	0.84	–0.39	0.80	–4.26^*^	2.84^*^
	RMIR	16.5 ± 0.57	15.91 ± 0.57	–0.46	/	0.03	–0.62	–0.14	–0.90	–0.83	–0.66	1.15	–0.77	0.23
	RHIR	**11.31 ± 0.38**	**10.54 ± 0.38**	1.59	/	–0.90	–0.76	–1.90	–1.52	–2.86^*^	–1.20	–1.24	0.52	–0.43

FW	RTD	106.95 ± 2.36	110.29 ± 2.1	0.15	0.76	1.72	1.36	2.65^*^	1.44	2.50^*^	/	0.90	–0.71	2.44
	RLIR	81.95 ± 1.74	82.1 ± 1.53	0.03	–2.22^*^	0.66	1.03	1.84	0.78	1.45	/	–0.75	0.41	1.88
	RMIR	16.56 ± 1.31	17.59 ± 1.15	0.13	4.88^*^	1.03	0.38	1.73	1.10	1.59	/	0.33	–1.18	0.76
	RHIR	**8.15 ± 0.59**	**9.83 ± 0.51**	0.18	0.53	1.59	0.45	2.67^*^	1.75	2.14^*^	/	1.14	–0.90	1.72

WM	RTD	**112.07 ± 2.57**	116.09 ± 2.56	0.08	–0.98	0.05	/	–0.51	–0.64	–0.20	/	1.74	–3.28^*^	2.87
	RLIR	84.42 ± 2.3	83.79 ± 2.23	1.35	–2.03^*^	–0.93	/	–1.17	–0.89	–0.59	/	–2.12^*^	–1.35	2.55
	RMIR	18.17 ± 1.88	19.26 ± 1.79	–0.49	1.57	1.09	/	0.54	0.18	0.74	/	3.15^*^	–1.15	1.32
	RHIR	**9.72 ± 0.68**	11.94 ± 0.66	–0.70	–0.17	0.55	/	0.48	–0.04	–0.06	/	3.10^*^	–1.93	–0.49

Note: CD – central defenders, OM – offensive midfielders, DM – defensive midfielders, FB – fullbacks, FW – forwards, WM – wingers; ML – match location, TF: 3241 – team formation 3-2-4-1; TF: 343 – team formation 3-4-3; TF: 352 – team formation 3-5-2; TF: 4231 – team formation 4-2-3-1; TF: 433 – team formation 4-3-3-, TF: 442F – team formation 4-4-2-flat, TF: 442D – team formation 4-4-2-diamond, OQ – quality of opposition, PT – playing time, RC – red card; RTD – relative total distance, RLIR – relative low-intensity running, RMIR – relative moderate-intensity running, RHIR – relative high-intensity running;

* – significant influence of fixed effect at p < 0.05. Bold text denotes significant differences in physical performance in won and lost matches at p < 0.05. Reference team formation for CD, OM, FB, and WM is 5-4-1. Reference formation for DM and FW is 4-4-2-D.

## DISCUSSION

This was the first study to examine position-specific physical performance when winning and losing in multiple teams that competed in the UCL while controlling for situational factors. The results demonstrated that winning was physically more demanding for offensive players but losing was more demanding for defensive players, while midfield players’ physical demands were similar irrespective of winning and losing. Therefore, the outcome of the match and playing positions should be considered when designing training programmes and developing specific game strategies in contemporary elite-level soccer.

It is commonly believed that winning is physically less demanding compared to losing [14, 15]. This is almost certainly due to the empirical evidence suggesting that winning is typically associated with high ball possession [16]. Theoretically, teams with high possession may show slower (i.e., positional) play, taking fewer risks when passing [17]. As a consequence, the team out of possession has to work harder physically to regain the ball. The results of the present study partially correspond to these observations, particularly with regard to defensive players, who showed increased workload when losing. Specifically, CDs covered slightly more LIR and RLIR in won than in lost matches (both medium ES). More importantly,

CDs performed ~ 15% less HIR and RHIR in won compared to lost matches (both large ES). Similar results were observed for FBs, who also covered more LIR and RLIR, and less HIR and RHIR in won versus lost matches (all medium ES).

Such an increased workload of defensive players when losing is almost certainly dictated by the opponent’s offensive players from the winning team [30] and can directly be supported by our results. Specifically, WMs and FWs accounted for slightly more TD and RTD in won compared to lost matches. More importantly, both WMs and FWs covered ~ 20% more HIR and RHIR in won than in lost matches (all large ES), which was most likely a consequence of their greater involvement in attacking activities [31]. As attacking activities are commonly executed at higher speeds [13, 32], defensive players of the opponent team are required to defend against these activities also utilizing higher speeds [33], which explains their increased workload when losing. Such findings are in line with previous studies investigating elite German Bundesliga players, reporting increased distance at higher speeds for both CDs and FBs in lost matches [5, 6].

Greater offensive players’ TD, RTD, HIR, and RHIR in won versus lost matches clearly suggest their increased workload when winning. As this contrasts with the common belief that winning is a comfortable status for a team and that therefore players’ workload is higher when losing than winning [14, 15], our findings may seem surprising. However, we believe that our findings are driven by the specific nature of the observed competition (i.e., UCL). Specifically, the current study investigated world-best soccer players [28], whose playing patterns may differ compared to the national-level players. For example, UCL players’ workload is greater [33] than that of national-level players [17, 34] when utilizing high-ball possession strategies. In this regard, UCL and national-level players may behave differently when winning as well, which may explain why our findings do not align with the common belief that losing results in a higher workload than winning [14, 15].

Although the roles of midfield players differ remarkably considering their positioning on the pitch, which consequently affects their physical performance [35], previous studies investigating physical performance according to the match outcome rarely separately analysed OMs and DMs. The current results indicated that OMs had more LIR and RLIR (both large ES), while DMs had more TD (large ES) and RTD (medium ES) in won compared to lost matches. At first, it seems that OMs’ and DMs’ physical demands differ when winning and losing. However, analysis of HIR and RHIR, which is a more suitable marker of physical demands due to its relationship with training status [36], indicated no differences for these parameters irrespective of winning or losing. This shows that both OMs and DMs experienced similar workloads in won and lost matches. It is most likely that midfielders maintained consistent roles and responsibilities on the field irrespective of the match outcome (i.e., controlling the tempo of the game, distributing the ball, supporting both defence and offense, maintaining possession, etc), possibly explaining their consistent running efforts both when winning and losing.

Several limitations should be noted when interpreting the findings of this study. Physical performance when winning and losing were assessed only by analysing speed distances, while acceleration frequencies, which are also an important parameter to assess physical demands [37], were not considered due to the unavailability of data. As only a limited number of situational factors that may affect physical performance were controlled for, future research should consider other factors such as effective playing time and/or weather conditions. On the other hand, this study also offers several strengths. This is the first time that physical performance in all matches through-out a single season of the UCL has been analysed; therefore, the results represent data unique to the literature. Moreover, the current study analysed physical performance separately for OM and DM, which has rarely been investigated before. Finally, this study is one of the first to provide a true effect of winning and losing on physical performance (i.e., by controlling for situational factors), offering valuable information to develop specific game strategies and training designs in contemporary soccer.

### Practical applications

It is noteworthy that increased offensive (when winning) and defensive (when losing) players’ workload was a result of increased distances covered at higher speeds, which imposes greater physiological and biomechanical demands on the body, leading to increased fatigue [38, 39]. This suggests that winning was physically more demanding for offensive players but losing was more demanding for defensive players. It is therefore imperative for soccer coaches to consider match outcomes and playing positions when designing training programmes and developing specific game strategies in contemporary elite-level soccer. Firstly, soccer coaches should consider extended periods of recovery or supplementary practices (e.g., ice submersions, massage) for offensive players after won matches and for defensive players after lost matches. This approach may efficiently improve players’ readiness for a subsequent training session and/or match. Secondly, substituting offensive players while winning and defensive players while losing should be considered earlier during the match as this may enable overall physical performance of the whole team to be maintained at the optimal level.

## CONCLUSIONS

The current study demonstrated the position-specific physical performance discriminating winning and losing in UCL. WMs and FWs covered more HIR and RHIR, with slightly increased TD and RTD, in won compared to lost matches. This suggests that offensive players experienced a greater workload when winning than when losing. In contrast, CDs and FBs had more HIR and RHIR in lost matches than in won matches, with slightly increased LIR and RLIR in won matches. This indicates that defensive players experienced a greater workload when losing than when winning. Contrary to the offensive and defensive players, OMs and DMs had similar physical performances in won and lost matches, showing that midfield players experienced similar workload irrespective of winning or losing. Finally, this study emphasizes the significance of taking into account match outcome and playing position in explanatory models when analysing physical performance in soccer, while at the same time reinforcing the importance of controlling for situational factors.
